# Design of the patient navigator to Reduce Readmissions (PArTNER) study: A pragmatic clinical effectiveness trial

**DOI:** 10.1016/j.conctc.2019.100420

**Published:** 2019-07-19

**Authors:** Valentin Prieto-Centurion, Sanjib Basu, Nina Bracken, Elizabeth Calhoun, Carolyn Dickens, Robert J. DiDomenico, Richard Gallardo, Victor Gordeuk, Melissa Gutierrez-Kapheim, Lewis L. Hsu, Sai Illendula, Min Joo, Uzma Kazmi, Amelia Mutso, A. Simon Pickard, Barry Pittendrigh, Jamie L. Sullivan, Mark Williams, Jerry A. Krishnan

**Affiliations:** a Breathe Chicago Center, Department of Medicine, College of Medicine, University of Illinois at Chicago, United States; b Department of Epidemiology and Biostatistics, School of Public Health, University of Illinois at Chicago, United States; c Population Health Sciences Program and Breathe Chicago Center, Department of Medicine, College of Medicine, University of Illinois at Chicago, United States; d University of Arizona, United States; e Department of Medicine, College of Medicine, University of Illinois at Chicago, United States; f Department of Pharmacy Practice, College of Pharmacy, University of Illinois at Chicago, United States; g Population Health Sciences Program, University of Illinois at Chicago, United States; h Department of Community Health Sciences, School of Public Health, University of Illinois at Chicago, United States; i Department of Pediatrics, College of Medicine, University of Illinois at Chicago, United States; j American Academy of Sleep Medicine, United States; k College of Medicine, University of Illinois at Chicago, United States; l Michigan State University, United States; m COPD Foundation, United States; n University of Kentucky, United States; o Population Health Sciences Program, And the Breathe Chicago Center, Department of Medicine, College of Medicine, University of Illinois at Chicago, United States

**Keywords:** Pragmatic clinical trial, Hospital-to-home transition, Hospital readmissions, Community health worker, Peer coaching

## Abstract

Previous work indicates the potential for community health workers and peer coaches serving as patient navigators to improve processes of care and health outcomes during care transitions, but have not been sufficiently tested to determine if such programs improve measures of patient experience in minority serving institutions. The objectives of the Patient Navigator to Reduce Readmissions (PArTNER) study was to: 1) conduct a pragmatic clinical effectiveness trial comparing a multi-faceted, stakeholder-supported Navigator intervention (in-person CHW visits in the hospital and after hospital discharge, plus telephone-based peer coaching) versus usual care on the experience of hospital-to-home care transitions in patients hospitalized with heart failure, pneumonia, chronic obstructive pulmonary disease, myocardial infarction, or sickle cell disease; 2) examine the effectiveness of the Navigator intervention in patient subgroups; and 3) understand the barriers and facilitators of successfully implementing the Navigator intervention across patient populations. The co-primary outcomes are the 30-day changes in: 1) Patient Reported Outcomes Measurement Information System (PROMIS) emotional distress-anxiety, and 2) PROMIS informational support. Secondary outcomes at 30 and 60 days include other PROMIS health measures and hospital readmissions. Innovative features of the PArTNER study include early and continuous engagement of patients, their caregivers, clinicians, health system administrators, and other stakeholders to inform the design and implementation of the Navigator intervention. In this report, we describe the design of the PArTNER study.

## Abbreviations

CHW: Community Health Worker
CMS: Centers For Medicare And Medicaid Services
COPD: Chronic Obstructive Pulmonary Disease
DPET: Discharge Patient Educational Tool
DSMB: Data And Safety Monitoring Board
EAC: External Advisory Committee
GED: General Educational Development
HIPAA: Health Insurance Portability And Accountability Act
IRB: Institutional Review Board
m: Missing
MAR: Missing at Random
MCID: Minimal Clinical Important Difference
MSI: Minority Serving Institution
NIH: National Institutes Of Health
NMAR: Not Missing at Random
PArTNER: Patient Navigator To Reduce Readmissions
PCORI: Patient Centered Outcomes Research Institute
PPA: Per-Protocol Analysis
PROMIS: Patient Reported Outcomes Measurement Information System
SD: Standard Deviation
UI Health: University of Illinois Health & Health Sciences System

## 1 Introduction

Staying healthy and avoiding unnecessary healthcare utilization is valued by patients and their caregivers. Their interests converge with those of hospitals now that higher-than-expected 30-day readmissions rates place hospitals at risk for financial penalties from the Centers for Medicare and Medicaid Services (CMS) and other payers [1]. Minority-serving institutions (MSIs), defined as institutions serving in the top 10th percentile of black and other minorities, have higher risks of adverse outcomes after hospital discharge, including readmissions and death, than other hospitals [2,3]. Additionally, some states are instituting financial penalties for higher-than-expected number of readmissions for Medicaid beneficiaries, a population disproportionately served by MSIs [[4], [5], [6], [7]]. These considerations indicate that MSIs will benefit from strategies tailored to the populations they serve, including Medicare and Medicaid beneficiaries [[8], [9], [10]].

There are several well-known evidence-based strategies to decrease avoidable readmissions, including Project Re-engineered Discharge (RED), Better Outcomes by Optimizing Safe Transitions (BOOST), and Care Transitions Program [[11], [12], [13]]. These strategies employ a range of interventions, including patient education, medication reconciliation, scheduling follow-up outpatient appointments to improve care, and care coordination. Patients and caregivers at MSIs have not historically played a major role in the development of such efforts, so not surprisingly these strategies emphasize interventions delivered by clinicians to improve process measures (e.g., care coordination, reducing polypharmacy) and reducing avoidable readmissions [[14], [15], [16]]. Recent literature suggests that reducing readmissions could inadvertently increase the risk of out-of-hospital deaths [17]. Moreover, patients and caregivers have expressed concerns that programs to reduce avoidable readmissions do not adequately address some critical aspects of the patient experience during care transitions, particularly concerns about abandonment, lack of confidence in knowing what to do, and anxiety [18].

Two previous single-center clinical trials in predominantly minority low-income populations examined the role of community health workers (CHWs; lay patient advocates from the community) as in person and telephone-based patient navigators for hospitalized populations suggest the potential to increase the proportion of patients who complete follow-up appointments after hospital discharge [19,20] and to reduce hospital-readmissions within 30 days [19].

Another clinical trial found that peer-to-peer coaching delivered by telephone to caregivers of children with asthma, including Medicaid beneficiaries, can increase symptom-free days and reduce healthcare utilization (emergency department visits and hospitalizations) [21]. While these findings are encouraging, none of these clinical trials or other studies included in a systematic review commissioned by the Agency for Healthcare Research and Quality specifically examined the role of CHWs and peer coaching by telephone in improving patient experience during hospital-to-home transitions as a primary outcome [22]. These considerations suggested the need for studies testing the effectiveness of strategies to improve patient experience during hospital-to-home transitions in high-risk Medicare and Medicaid beneficiaries at MSIs.

The overall goal of the Patient Navigator to Reduce Readmissions (PArTNER) study was to conduct a randomized 2-arm parallel group, pragmatic single-center clinical trial comparing the effectiveness of CHWs and peer coaches (Navigator intervention) versus usual care to improve patient experience during hospital-to-home transitions at a MSI. The focus of the PArTNER study consisted of medical populations targeted by CMS penalties to reduce avoidable readmissions (hospitalizations for heart failure, pneumonia, chronic obstructive pulmonary disease, and myocardial infarction) and populations who disproportionately contribute to penalties among Medicaid beneficiaries at MSIs (hospitalization for sickle cell disease) [[23], [24], [25]]. The objective of this report is to describe the design of the PArTNER study and its innovative features, including the integration of existing patient advocacy organizations to deliver the Navigator intervention.

## 2 Materials and methods

### 2.1 Study overview and design

**Fig. 1:**
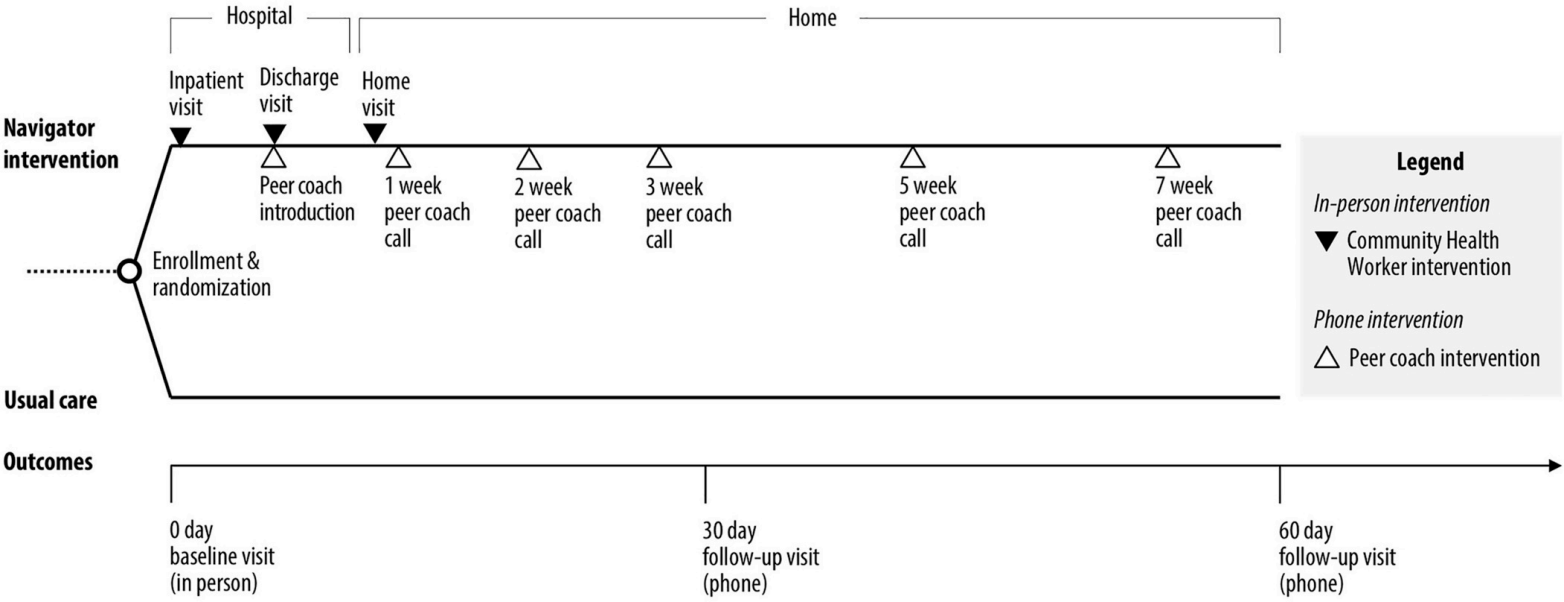
PArTNER study design. In the PArTNER study, participants hospitalized with a physician diagnosis of heart failure, pneumonia, chronic obstructive pulmonary disease (COPD), myocardial infarction, or sickle cell disease are randomly allocated to one of two groups: Navigator intervention or Usual care. The Navigator intervention spanned over a 2-month period after hospital discharge and included: 1) community health workers (CHWs) who conducted in-person study visits in the hospital and a single home visit 1–3 days post-discharge to assess barriers to patient-centered transitions from hospital to home, and 2) peer coaches are introduced on hospital discharge and contact participants via telephone at approximately 1, 2, 3, 5, and 7 weeks post-discharge to continue supports initiated by CHWs. Following in-person baseline data collection prior to randomization, follow-up outcomes were assessed via telephone at 30 days and 60 days post-discharge.

**Fig. 2:**
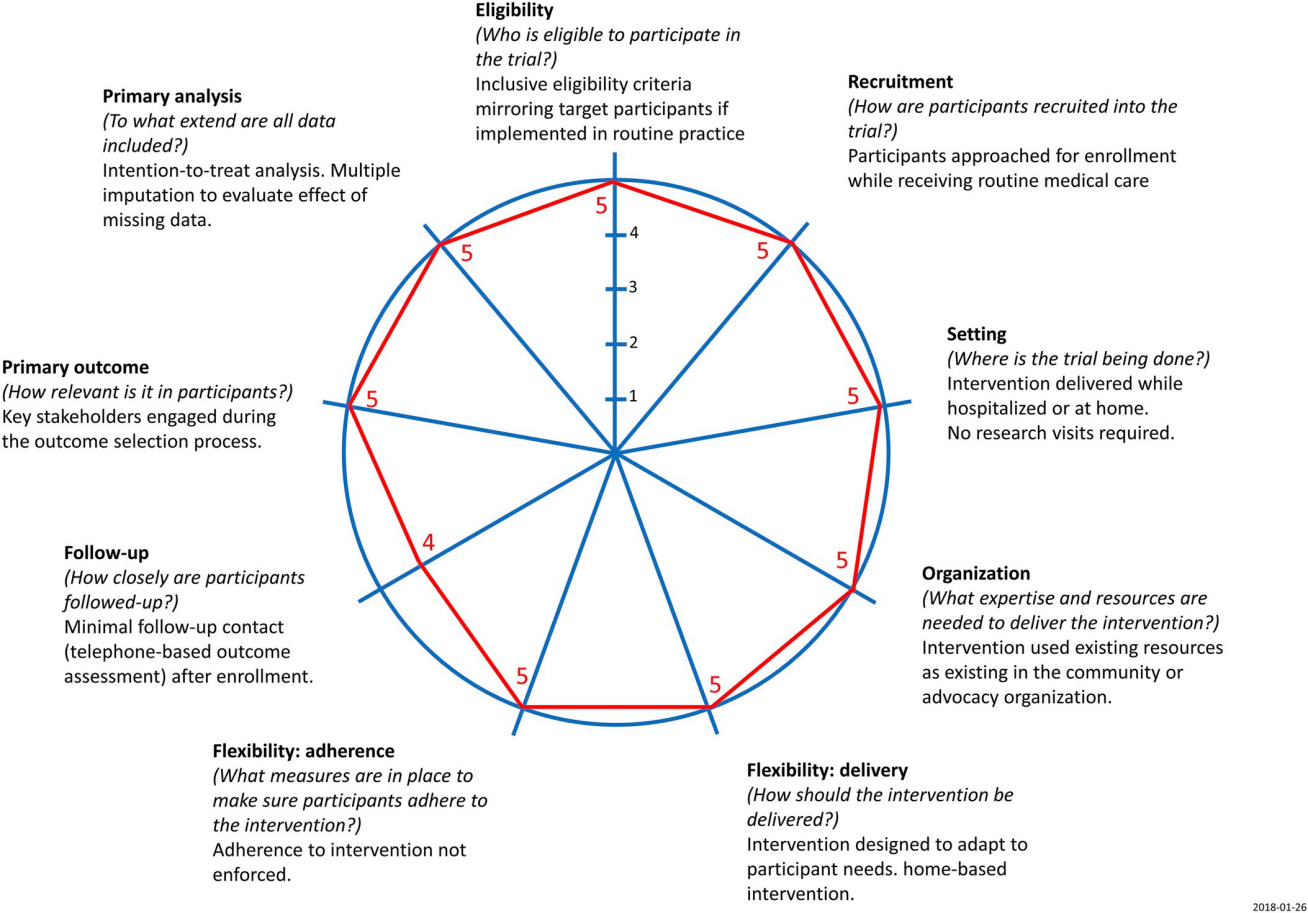
Pragmatic design features of the PArTNER study according to the PRECIS-2 instrument. The study design features were based on input from patients, caregivers, clinicians, and hospital administrators during the planning stages of the PArTNER trial; goal was to develop a study consistent with a pragmatic (effectiveness) trial [26]. The “follow-up” category was graded as a 4 out of 5 as participants were contacted by phone to outcome data, rather than relying on passive approaches to data collection. Passive appraoches to data collection for assessing patient-reported measures of physical, emotional, and social health are not currently available.

The PArTNER study is a two-arm randomized controlled pragmatic clinical effectiveness trial comparing the Navigator intervention to Usual care (Fig. 1). Several aspects of the study design are intended to reflect a priority to generate evidence closer to the “effectiveness” end of the continuum between efficacy (i.e., ideal conditions) and effectiveness (i.e., real world conditions) to address needs identified by end-users and a strategy that would be feasible to implement after the end of the study period (Fig. 2) [26].

### 2.2 Participants

Patients hospitalized at UI Health (see Study setting, Section B.6), with a physician diagnosis of heart failure, pneumonia, COPD, myocardial infarction, or sickle cell disease are eligible for study enrollment. Trained research coordinators will use IRB-approved recruitment strategies to screen electronic health records Monday through Saturday to identify hospitalized patients with the target diagnoses. Research coordinators will contact the treating physicians to confirm the admission diagnosis documented in the electronic health record and to obtain verbal assent to approach the patient for enrollment into the study. Written informed consent will be obtained from all participants prior to enrollment. To be eligible, patients need to meet all inclusion criteria and none of the exclusion criteria described in Table 1.

**Table 1:** PArTNER study inclusion and exclusion criteria.

Inclusion criteria	Exclusion criteria
1. Hospitalized at UI Health	1.Unable to understand and/or speak English
2. Aged 18 years or older	2. Unable/or declined to give informed consent
3. Index admission physician diagnosis of: - Heart failure, - Pneumonia, - Chronic obstructive pulmonary disease, - Myocardial infarction, or - Sickle cell disease	3. Previous participant in the PArTNER study*
	4. Planned transfer to another acute care facility
	5. Planned discharge to facility other than home (e.g., long term care facility)
	6. Receiving hospice care or planned to be discharged to hospice care
	7. Plan to leave against medical advice

Footer: UI Health – University of Illinois Hospital & Health Sciences System; * refers to patients who were re-hospitalized following completion of participation in the PArTNER study.

**Table 2:** Community health worker (CHW)-led interventions implemented in the PArTNER study.

Component of CHW-led intervention	Description
A. Complete barrier assessment linked to socioeconomic resources	• Assessment of need for assistance with employment and income, family and social support, transportation to healthcare facilities, housing, utilities (e.g., heat, light), food, and interpersonal violence. • Barriers selected based on the Society of Hospital Medicine's Project BOOST,^16^ and interviews with stakeholders (patients, caregivers, clinicians, administrators at UI Health). • As appropriate, review barriers with the participant's clinician and social worker.
B. Offer and assist with participant-specific needs by helping patients to identify and receive assistance from resources	• Development of a tailored, patient-centered plan for solutions to each identified barrier. Resources included those available through Purple Binder (website that houses information for medical and social service resources in the Chicago area) and those used by the hospital's social work department. • Review of solutions with the participant during subsequent visits and/or through email and text messages. • Reassessment of barriers and identification of new ones during each CHW in-hospital encounter. • Caregivers included in the discussions if requested by participant.
C. Completion of a “Discharge Patient Education Tool”	• Completion of personalized discharge patient educational tool (DPET) based on discharge instructions in the electronic health records. • Core content areas reviewed with participant using teach-back: - Post-discharge follow up visits and tests, - Recommendations regarding lifestyle changes, and - Use of medications after discharge. • CHW scheduled post-discharge follow-up appointments as needed. • Review of previously-identified barriers and potential solutions, including post-discharge resources and services. • Scheduling of home visit of CHW within 3 business days of hospital discharge. • Referral to the participant's clinicians for medical advice. - The CHWs were specifically instructed to not provide medical advice.
D. Re-review the DPET with the participant	• During home visit, review of DPET with the participant, including: - Reminder of upcoming tests and appointments - Confirmation of availability of transportation to tests and appointments - Review understanding and adherence to lifestyle changes - Confirmation of availability of medications - Confirmation of understanding and adherence to medications • If the participant had difficulty adhering to the DPET, peer coached identified new barriers and worked with participant to find a solution. • Participants encouraged to contact the hospital's social worker or clinician's office, if needed.
E. Re-review solutions to barriers with patient	• Review solutions implemented for previously identified barriers. • If needed, identification of new barriers and potential solutions
F. Peer coaching	• Introduction of peer-coaching intervention prior to hospital discharge. • Reminder about upcoming phone-based peer coaching calls during the home visit. • Peer coaches had access to barriers and solutions identified by CHW through study data system.

**Table 3:** Peer coach intervention components.

Peer Coach Component	Description
A. Greeting and Reminder	IRB-approved script used to confirm the participant's identity and remind them of their participation in the study.
B. Review of the participant's DPET.	• Review of DPET with the participant, including: - Reminder of upcoming tests and appointments - Confirmation of availability of transportation to tests and appointments - Review understanding and adherence to lifestyle changes - Confirmation of availability of medications - Confirmation of understanding and adherence to medications • If the participant had difficulty adhering to the DPET, peer coached identified new barriers and worked with participant to find a solution.
C. Review previous barriers and solutions.	• Review of previously-identified barriers and potential solutions • Identification of new barriers and potential solutions (similar procedure as CHW)
D. Schedule next peer coaching intervention call.	• Schedule next (of five) peer coaching intervention call: - Participants offered flexibility in scheduling according to their availability - Last call occurred by 60 days post-discharge.

### 2.3 Randomization

Following baseline data collection, we will employ block-stratified randomization with permuted blocks to promote balance in the number of participants in each of the two PArTNER study groups (Navigator intervention and Usual care) in three key baseline participant characteristics: 1) enrollment condition (heart failure, pneumonia, COPD, myocardial infarction, or sickle cell disease); 2) baseline anxiety (Patient Reported Outcomes Measurement Information System [PROMIS] emotional distress-anxiety v1.0, SF4a,T-score ≥50, yes/no); and 3) baseline informational support (PROMIS informational support, v2.0, SF4a, T-score ≥50, yes/no) [27]. The random allocation sequence will be generated by a computerized random number generator. Strata will include baseline values of anxiety (PROMIS emotional distress-anxiety) and informational support (PROMIS informational support), since changes from baseline values for emotional distress-anxiety and informational support at 30 days were the co-primary outcomes. Research staff will be masked to treatment allocation sequence prior to informed consent and baseline data collection as our data system required this information to generate a treatment allocation. Research staff collecting post-randomization outcomes will also be masked to treatment allocation as they do not have access to treatment-related information.

### 2.4 Power and sample size

Assuming 20% attrition and a Bonferroni correction for two co-primary outcomes (2-sided alpha 0.025), we estimate that 1,130 participants (565 participants in each treatment group) are needed for 95% or greater power to detect a 2.5 unit difference (25% of a standard deviation, or T-score of 2.5) for each of the co-primary outcomes in the overall population. The minimum detectable difference in T-score of 2.5 falls well within the T-score minimal clinical important difference (MCID) of 2–5. The 95% power for the primary outcomes was selected to allow the calculated sample size to also provide 80% power to detect a 7.5% absolute reduction in 30-day risk of death or re-hospitalization (from 30% to 22.5%) and 90% power to detect a 10% reduction.

### 2.5 Intervention and comparator

#### 2.5.1 Navigator intervention

All participants (regardless of treatment group) will receive usual healthcare as per their treating medical team (see section B.5.2).

The Navigator intervention was designed with input from multiple stakeholder groups, including patients, their caregivers, front-line hospital-based and ambulatory clinicians (hospitalists, primary care, cardiology, hematology, pulmonary, social work), and hospital administration (quality improvement and population health leadership) [18]. Stakeholder engagement was used to ensure the Navigator intervention directly addressed end-user requirements and would be feasible to implement. The intervention will be delivered by CHWs and peer coaches during the index hospitalization and continued for two months after hospital discharge. The two CHWs for the PArTNER study (both women; one Latino who was bilingual, Spanish and English; the second was black and spoke English) were carefully selected to have at least a General Educational Development (GED) or high-school diploma, a passion for working in the communities served by UI Health (primarily west and southside Chicago), and a valid driver's license. The CHWs received education from investigators and staff on the health conditions of interest, techniques for approaching and connecting with patients in the hospital setting, safety during home visits, and appropriate documentation of interactions with study participants. The project manager for the study was responsible for daily supervision, with the help of a nurse practitioner and physicians on the study team.

The peer coaches are staff at existing patient advocacy organizations (Section B.5.1.2). Peer coaches are required to have a telephone and internet access at home to facilitate training, data entry, and rapidly retrieving information about the communities in which patient lived. Peer coaches underwent training that included a 45-h web training session on 1) customer service and call etiquette, 2) the Health Insurance Portability and Accountability Act (HIPAA), 3) basic training about the health conditions that formed the target population, 4) program-specific training for PArTNER and how to find community resources (e.g., food pantry, transportation, housing), and 5) use of a HIPAA-compliant portal to document all calls. The curriculum was based on training that was used by the COPD Foundation for its call-center and supplemented with information relevant to the PArTNER study. The number of peer coaches varied during the course of the PArTNER study but at least ten peer coaches were involved in the study at any particular point. The CHW and peer coaches were instructed to use a HIPAA-compliant web-based database to record interventions completed for each study participant.

##### 2.5.1.1 CHW-led interventions (Table 2)

The CHWs interviewed participants in person during the index hospitalization and at a home visit within three days of hospital discharge. The goal of these CHW-led sessions was to identify barriers to health and healthcare and to provide support to promote self-management skills. Potential resources for participants will be accessed using an interactive online resources database that allowed identification of hyper-local resources to each unique participant [28]. The CHW intervention is flexible to allow it to occur via phone or around the time of follow-up appointments at UI Health if needed. The initial visit is scheduled to occur over approximately 45 min. The number of visits conducted by the CHW during hospitalization is dependent on participants’ needs and length of stay; participants will receive daily visits in some cases.

##### 2.5.1.2 Peer-coach-led interventions (Table 3)

The telephone-based peer coaching support at weeks 1 (intervention window 6–10 days), 2 (12–16 days), 3 (19–23 days), 5 (32–37 days), and 7 (47–51 days) after hospital discharge is intended to continue supports initiated by the CHW intervention (Fig. 1). Each peer coach call is scheduled for approximately 15 min, while each participant could have had more than one peer coach across the five calls (mimicking real-world circumstances). Patient advocacy organizations will provide peer coaching support for participants hospitalized with pneumonia or COPD (COPD Foundation), myocardial infarction or heart failure (Mended Hearts), and sickle cell disease (Sickle Cell Disease Association of Illinois) [[29], [30], [31]]. The “peer coaching” intervention by phone is intended to leverage existing services and to connect participants with resources in the community. The COPD Foundation has a well-developed peer coaching (patients with COPD or caregivers of patients with COPD) infrastructure that provides a method for tracking and recording peer coaching phone calls. If a peer coach is not available from the specified organization, the CHW would temporarily fill-in to complete peer coaching calls. Through the HIPAA-secure database, peer coaches have access to the CHW-completed *Barrier Assessments* and *Discharge Patient Educational Tool (DPET*; see online supplement) that is specific to their patients, helping to facilitate a team response and to know each participant's unique needs. Over the course of approximately 60 days post-discharge, peer coaches will use a sequence of up to five phone calls to provide support in addressing barriers to care and the use of the DPET to promote self-management. Up to four call attempts will be made for each peer coaching call (will attempt to leave voicemails after each unsuccessful call attempt).

#### 2.5.2 Usual care

Because usual hospital-to-home transitional care can vary within and across institutions, we plan to review the discharge documentation in the electronic health record for all participants (regardless of treatment group) to evaluate care provided by the clinical teams. This review will be conducted by study staff who are masked to treatment allocation sequence. We used stakeholder input and the framework proposed by the Society of Hospital Medicine's BOOST Program (“universal discharge checklist”) to define the eight transitional care elements of interest in describing usual care (yes or no; Table 4) [12]. Front-line clinicians who provide care to patients with target conditions for the PArTNER study agreed with the definitions for each of eight transitional care indicators in Table 4. In a random 10% sample of study participants, a second research coordinator will repeat the data abstraction to assess inter-rater agreement (kappa statistic). All discrepancies will be reviewed, adjudicated, and corrected values will be entered into the study database.

**Table 4:** Description of Universal Discharge Components used in the PArTNER study to evaluate care provided by the participant's clinical team.

Universal Discharge Component	Description
A. Medication reconciliation performed on the date of discharge	Medication reconciliation marked as complete and/or a pharmacist note documenting medication reconciliation in the electronic health record.
B. Medication education provided to patient on date of discharge	Educational materials specific to a class of medications, a specific medication, or device (e.g., respiratory inhaler, oxygen equipment) provided to patient and/or pharmacist note documenting the education in the electronic health record.
C. Education on diagnosis, prognosis, self-care requirements, or procedures provided to patient on the date of discharge	Educational materials for any medical conditions listed in the discharge summary is documented in the electronic health record's Patient Education Note.
D. Disease management education provided to patient on the date of discharge	Instructions about how to seek additional care in case of clinical deterioration, or specific to the patient's medical condition recorded in the electronic health record.
E. Structured Discharge Summary completed upon hospital discharge:	Discharge summary recorded as finalized by the attending physician in the electronic health record within two [2] business days of hospital discharge.
F. Phone contact attempted within two business days post discharge with patient or caregiver.	Call attempt documented in the electronic health record as successful or unsuccessful within two [2] business days after hospital discharge.
G. Follow-up appointment within seven days of hospital discharge is specified in discharge instructions	Appointment information (at a minimum: location, date, time) in the electronic health record with at least one provider scheduled for seven or fewer days after hospital discharge; the provider location could be outside of the index hospital-affiliated clinics.
H. Discharge instructions provided to patient on the date of discharge	Documentation in the electronic health record that discharge instructions were provided to patient on the same date as discharge.

Footer: Study staff masked to treatment group assignment reviewed the electronic health record for all study participants to assess the presence or absence of each discharge component (see Methods for details).

### 2.6 Study setting

The PArTNER study is a single-center clinical trial that enrolls patients hospitalized at UI Health. UI Health hospital is the largest state-supported hospital in Illinois and includes a 495-bed tertiary care hospital, 22 outpatient clinics (primary care and specialty care), and 11 federally qualified health centers. UI Health is a MSI (hospitalized patients are ∼80% Black) and represents an ideal environment to evaluate the effectiveness of care transition services tailored to this high-risk population.

### 2.7 Time frame for the study

The intervention will span over a two-month period after hospital discharge (Fig. 1). Outcomes will be assessed at 30 days and 60 days. This outcome assessment time frame was selected not only to inform patients, caregivers, and other decision-makers about the potential of the Navigator interventions to address short-term measures of health on completion of the intervention (60 days), but also to provide information on the effect of the intervention on outcomes of interest to payers and health systems administrators, such as 30-day readmissions and deaths. There was insufficient stakeholder support for a longer (and more resource intensive) intervention and follow-up period.

### 2.8 Data collection and sources

The ascertainment of outcomes after hospital discharge will be performed by a trained research coordinator who is masked to treatment group allocation. The research coordinator will attempt to contact participants by telephone. Up to 14 call attempts will be made to collect patient-reported outcomes for each of the 30- and 60-day assessments. Electronic health records will also be reviewed to assess outcomes, particularly healthcare utilization, at 30- and 60-days post-discharge.

Many aspects of our study design are intended to reduce the frequency of missing data. We designed the study to target a population not adequately served by current services, which could help serve as an incentive for clinicians to support the study and for participants to remain in the study. We will accommodate individual preferences as much as possible when setting follow-up visits or data collection time-points. The study enrollment and data collection will be performed as participants receive routine care in the hospital and with reduced burden post-discharge (e.g., data collection by phone, rather than in-person). Also, we do not equate treatment discontinuation with study drop-out; we will encourage participants to continue in the study and provide outcomes data even if they no longer wish to remain in the treatment group assigned by randomization. We will also encourage participants to continue in the study by explaining the importance of completing the study with as many participants as were enrolled to avoid selection bias. Reasons for withdrawing from the study or lost to follow-up will be collected when possible by interviewing study participants, caregivers, and review of electronic health records (e.g., participant died), and reporting the information in a participant flow diagram.

### 2.9 Data and safety monitoring

Study procedures and updates will be reviewed during meetings with an independent Data and Safety Monitoring Board (DSMB) with 5 members: 3 physicians (2 hospitalists, 1 geriatrician), 1 pharmacist, and 1 nurse who provide direct patient care. The DSMB will be asked to review and approve the study protocol prior to initiation of participant recruitment and to convene twice yearly to review the study progress and make recommendations, including continuing study without modifications, continuing study with modifications, and terminating the study. We will submit draft written agendas prior to meetings and written summaries of discussions and next steps after each meeting.

### 2.10 Study outcomes

#### 2.10.1 Primary outcomes

Patient and caregiver engagement activities indicated the need for two co-primary outcomes for the clinical trial:

1. Change in participant anxiety from baseline (enrollment to 30 days post-discharge).
2. Change in participant informational support (enrollment to 30 days post-discharge).

Informational support refers to the ability to obtain advice, guidance, suggestions, or useful information.

We will use the National Institutes of Health (NIH) PROMIS measures for our co-primary outcomes, since the development and validation of PROMIS scales have been extensively documented [27]. The PROMIS emotional distress-anxiety short form (version 1.0 SF4a, 4 items) will be used to assess participant anxiety in the previous 7 days. The PROMIS informational support short form (version 2.0 SF4a, 4 items) will be used to assess informational support among study participants in the previous 7 days. For PROMIS measures, raw scores for each measure will be re-scaled into a standardized T-score with a mean of 50 and standard deviation of 10 for the US general population [27,32,33]. Higher PROMIS T-scores indicate more of the concept being measured (e.g., higher anxiety T-scores indicate more anxiety). Studies suggest that a 2- to 5-unit or greater difference in T-scores is likely to be the MCID, though the MCID following hospital discharge in the target population has not been established.

Using PROMIS measures confers several distinct advantages: 1) *Comparability*—measures have been standardized to be patient-centered, rather than disease-specific, permitting comparisons of patient outcomes between the Navigator and Usual care groups despite enrolling patients with multiple conditions; 2) *Reliability* and *Validity*—metrics for each domain have been rigorously reviewed and tested; 3) *Flexibility*—PROMIS measures can be administered in a variety of ways (in person, telephone, or via computer adaptive testing); for PArTNER, the baseline visit occurred while the patient was in the hospital, so administration was in person. Follow-up visits were performed by telephone to minimize participant burden (a key principle in effectiveness trials); and 4) *Inclusiveness*—PROMIS encompasses all people, regardless of literacy, language, physical function, or life course.

#### 2.10.2 Secondary outcomes

Secondary outcomes include assessments of change in participant PROMIS anxiety and in informational support T-scores from enrollment to 60 days after hospital discharge, as well as changes of other PROMIS measures (instrumental support [version 2.0, SF4a, 4 items; emotional support [version 2.0 SF4a, 4 items], mental health [version 1.1 SF, 4 items within the 10-item global health scale], physical health [version 1.1 SF, 4 items within the 10-item global health scale]) from enrollment to 30 and 60 days after [27].

We will also assess healthcare utilization as secondary outcomes. We will assess attendance at outpatient clinics within 14 days of hospital discharge (using self-report; and separately using review of electronic health records at UI Health, which is shared with the hospital and all ambulatory clinics within health system, including a network of 11 federally qualified healthcare centers); death at 30 and 60 days after hospital discharge (based on caregiver report or review of electronic health records); rehospitalization at 30 and 60 days (assessed by review of electronic health records); emergency department visit at 30 and 60 days (assessed by review of electronic health records); death or re-hospitalization at 30 and 60 days; and finally, death or re-hospitalization or emergency department visit at 30 and 60 days. We do not have access to claims data to ascertain healthcare outside of the hospital or ambulatory clinics outside UI Health.

### 2.11 Analytical and statistical approaches

Analyses will be performed in three steps. *Step 1* consists of exploratory analyses to identify, correct, and confirm values or missing data, and provide descriptive statistics (frequency [proportions], mean [standard deviations]). *Step 2* focuses on bivariate analyses to compare primary and secondary outcomes by treatment group. We will use t-tests, Wilcoxon rank-sum tests, chi-squared tests, and Fisher's exact tests, as appropriate, for pairwise comparisons between the Navigator and Usual care groups. *Step 3* includes multivariable logistic or linear regression models, as appropriate, to account for potential confounders for the primary and secondary outcomes. The co-primary analysis is pre-specified as use of multivariable linear regression models comparing change in anxiety and change in informational support from enrollment to 30 days after hospital discharge in the Navigator group compared with the Usual care group, after adjusting for potential confounders (i.e., adjusted differences). We pre-defined potential confounders as baseline age (>65 years, yes/no), gender (women vs. men), race (non-Hispanic black vs. other), target health condition (pneumonia, heart failure, COPD, myocardial infarction, sickle cell disease), having a primary care provider (yes/no), patient-reported usual source of healthcare (doctor's office, emergency department, community health center, other), patient-reported hospitalization in the 12 months prior to index hospitalization (yes/no), education (high school or less vs. other), and health insurance (yes/no). To minimize the risk of bias in specifying potential confounders, we will examine the baseline characteristics of study participants while being masked to treatment group allocation before finalizing the list of potential confounders for the multivariable analyses.

All analyses will be conducted using a modified intention-to-treat principle, in which we ignored the individual with the missing data for the co-primary outcomes (complete-case analyses). To assess the sensitivity of results to the complete-case analyses, we will conduct multiple imputations under a missing at random (MAR) assumption [34]. We will also conduct multiple imputations under a missing not at random (MNAR) assumption under a joint selection model of outcome and missingness and employ Bayesian analyses for each of the two co-primary outcomes. Our multi-tiered approach to missing data will be used to determine if the Navigator intervention effects are qualitatively maintained across various approaches to handling missing data. We will also conduct per-protocol analyses (PPAs) for the primary outcome. In the PPAs, the Navigator intervention will be considered per-protocol if the participant received both hospital and home-based components of the CHW-led interventions and at least one peer-coaching call. We will employ a 2-sided Bonferroni-corrected alpha of 0.025 (and 97.5% confidence intervals, CIs) for the two co-primary outcomes in the multivariable analyses, and a 2-sided alpha of 0.05 (and 95% CIs) for all other hypothesis tests.

We will also conduct exploratory analyses to assess the potential for heterogeneity of treatment effects for the primary outcome by examining the consistency of the adjusted differences in pre-specified subgroups (across levels of each baseline characteristic) in a series of linear regression models with covariates that included the treatment-group indicator, the treatment-by-subgroup interaction terms, and all other covariates in the multivariable models. We will also collect a limited number of baseline characteristics in keeping with information that is routinely collected on hospitalizations and that have been associated with hospital readmissions at MSIs [35]. The p-values for consistency of adjusted differences in linear or logistic regression models across subgroups will be determined by Wald chi-squared tests. The PArTNER study was not specifically powered to assess heterogeneity of treatment effects, so all such analyses should be considered exploratory. Due to multiple hypothesis tests, we will employ Bonferroni corrections to reduce the risk of a type 1 error.

A limitation of research directed at improving health systems is that there is often inadequate attention devoted to understanding why an intervention was successful or not successful. Health system interventions are often multi-component, and when they successfully improve care or outcomes, it is helpful to know whether all components of the intervention were necessary for success. Also, when care or outcomes are not improved, it is unclear if barriers to implementation (fidelity) or lack of efficacy contributed to a lack of effect. We therefore will implement a mixed-methods approach to: 1) assess completion of each component of the multi-component Navigator intervention (completion of the barrier assessment, discharge visit, home visit, peer coaching calls) and 2) conduct interviews to debrief with study staff and a convenience sample of participants spanning all five enrollment conditions.

## 3 Discussion

Although early hospital readmission has recently gained prominence with reimbursement models that include financial penalties to health systems with excess 30-day readmissions by CMS and other payers, the patient and caregiver experience during hospital-to-home transitions had not been extensively studied. MSIs provide a disproportionate share of care to patients who have especially high risk of early hospital readmission, which also tend to be patients with lower socioeconomic resources. Among Medicare beneficiaries, readmission rates are significantly higher for African Americans at MSIs (26%) than whites at non–MSIs (21%) (2,37) These findings suggest that financial penalties for excess hospital readmissions are likely to disproportionately affect MSIs, further limiting their available resources to care for this population. This highlights the need to develop and evaluate low-burden interventions that, if shown to be effective, have the potential for widespread implementation.

Innovative aspects of the PArTNER study include its focus on the patient experience during the hospital-to-home transition, early and continuous stakeholder engagement to inform the design and implementation of the study, and integrating patient advocacy organizations in the delivery of the Navigator intervention (peer coaching).

Results of the stakeholder engagement phase were used to ensure that the Navigator intervention was tailored to the local needs of end-users, including patients, caregivers, front-line clinicians, and health system administrators, and feasible to implement during routine healthcare operations after the completion of the study period.

The study outcomes were selected to address the needs of the multiple stakeholders involved in the design and implementation of the PArTNER study. The primary outcomes were selected to address patient preferences to focus on two measures of patient experience (patient-reported anxiety and informational support). These co-primary outcomes were selected as they are mechanistically directly linked to the intervention procedures, that is, to provide social and informational support. Some of the secondary outcomes, such as the different measures of healthcare utilization, were selected to address the needs by health system administrators regarding unplanned early readmissions. The method of outcome collection, including collection of baseline measures while participants are hospitalized and telephone-based collection of follow-up outcomes, and limiting the number of outcomes that were collected, were designed to minimize participant burden.

The focus on several CMS penalty-sensitive conditions for hospital readmissions (heart failure, pneumonia, COPD, myocardial infarction, and sickle cell disease, a condition with among the highest readmission rates among Medicaid beneficiaries) is potentially relevant to many health systems in the U.S. However, our study was conducted in a single, state-supported MSI with relatively good transitional care services; these factors may limit the external validity of the study findings.

Given the complexity of the Navigator intervention, it is possible that some aspects of the intervention (especially peer coaching) will not be completely implemented in all participants. Although process measures, such as fidelity of implementation of the intervention, will be collected by the study staff, it may not be possible to establish a clear relationship between participant outcomes and the “dose” of the intervention they received. As the intervention is specifically designed to target participants with low socioeconomic resources, another potential limitation is missing outcome data due to inability to reach participants due to unstable housing or telephone contact information.

In summary, the PArTNER study will evaluate the effectiveness of a Navigator intervention led by CHWs and peer coaches that target social determinants of health on stakeholder-supported measures of patient experience and clinical outcomes in patients hospitalized at an MSI. We hope the lessons from the PArTNER study will be of interest to patients and their caregivers, hospital leaders and staff who plan and implement hospital-to-home transitional care services at MSIs, as well as payers and policymakers interested in promoting more effective care transitions in high-risk populations.

## Funding sources

The PArTNER Study was funded through a Patient Centered Outcomes Research Institute (PCORI) award (contract # IH 12-11-4365; Clinicaltrails.gov registration #NCT02114515). The statements in this report are solely the responsibility of the authors and do not necessarily represent the views of PCORI, the PCORI Board of Governors, or the PCORI Methodology Committee.
