# Supplementary material for: Design of the patient navigator to Reduce Readmissions (PArTNER) study: A pragmatic clinical effectiveness trial
Source: Contemp Clin Trials Commun. 2019 Jul 19;15:100420. doi: 10.1016/j.conctc.2019.100420 (PMC6700266; doi:10.1016/j.conctc.2019.100420)
Supplement: Multimedia component 1 [file mmc1.pdf]

Design of the Patient Navigator to Reduce Readmissions (PArTNER) study:

A pragmatic clinical effectiveness trial

Online Data Supplement

Valentin Prieto-Centurion, MD, MS[1]; Sanjib Basu, MStat, PhD[2]; Nina Bracken, APRN[3]; Elizabeth Calhoun, PhD[4]; Carolyn Dickens, PhD, APRN[1]; Robert J. DiDomenico, PharmD[5]; Richard Gallardo, MPH[3]; Victor Gordeuk, MD[1]; Melissa Gutierrez-Kapheim, MS[6]; Lewis L Hsu, MD, PhD[7]; Sai Illendula, MS[3]; Min Joo, MD, MPH[1]; Uzma Kazmi, MPH[8]; Amelia Mutso, PhD[1]; A. Simon Pickard, PhD[5]; Barry Pittendrigh, PhD[9]; Jamie L. Sullivan, MPH[10]; Mark Williams, MD[11]; Jerry A. Krishnan, MD, PhD[1,3]

1. Department of Medicine, College of Medicine, University of Illinois at Chicago
2. Department of Epidemiology and Biostatistics, School of Public Health, University of Illinois at Chicago
3. Population Health Sciences Program, University of Illinois at Chicago
4. University of Arizona
5. Department of Pharmacy Practice, College of Pharmacy, University of Illinois at Chicago
6. Department of Community Health Sciences, School of Public Health, University of Illinois at Chicago
7. Department of Pediatrics, College of Medicine, University of Illinois at Chicago
8. American Academy of Sleep Medicine
9. Michigan State University
10. COPD Foundation
11. University of Kentucky

February 14, 2019

Transportation from hospital

Remember to make transportation plans to and from your next doctor's appointments.

For

Being picked up by

Phone #

Going to

At address

Illinois

Alternate contact(s)

Emergency information for

If you need immediate help, call 9-1-1 or go to the nearest emergency department or urgent care facility.

Watch for signs + symptoms

-
-
-
-
-
-

Take action

Call
at if
has the symptoms above

Continues on back

Post this page where it is easy to see in case of an emergency.

Get updated information from your follow-up doctor at each follow-up appointment.

Other doctor's information

Doctor's name:

Type of doctor:

Doctor's phone #:

Hospitalization summary

▶ Be sure to give this information when you call

Patient name:

Medical record #:

Date of birth:

Admission date:

Discharge date:

Primary discharge diagnosis:

Insurance information

Insurance type:

Insurance #:

Insurance phone #:

Insurance care coordinator:

1 Learn about the PArTNER study + UI Health

What's inside

1A PArTNER study overview

1B UI Health community clinics

Consent form

This information will help you...

- understand the study you enrolled in
- know what clinics are available in the UI Health system

1A *Learn about the PArTNER study + UI Health*
PArTNER study overview

The problem

Many patients return to the hospital less than a month after they are discharged from the hospital.

It can be hard for patients to get back on their feet after they return home.

During this time, patients sometimes feel abandonment, anxiety, fear, or as if they can't take care of themselves as well as they would like.

PArTNER's mission

The PArTNER study is trying to find out
- what patients need when they go home
- how hospitals can help them stay healthy during this time

Your role in PArTNER

After you are discharged home, you will get:

2 calls from a research coordinator.

They will ask you questions about your current health.

These calls last around 15 minutes

1A *Learn about the PArTNER study + UI Health*

PArTNER study overview

Your navigator + peer coach

In addition to the 2 calls from a research coordinator, you will get:

1 home visit from your **patient navigator**

Your patient navigator helps to make sure you are able to stay healthy once you leave the hospital.

Patient navigators

Dameka Edwards	Emma Villarreal
1.312.636.3449	1.312.636.3429

5 calls from your **peer coach (iPArTNER)** over the next 2 months. Each call lasts around 15 minutes.

Your peer coach has a condition similar to yours. They are members of one of these organizations:

- COPD Foundation
- Mended Hearts
- Sickle Cell Disease Association of Illinois

Peer coach (iPArTNER)

1.844.iPrTNER
(1.844.477.8637)

1B

Learn about the PArTNER study + UI Health

UI Health community clinics

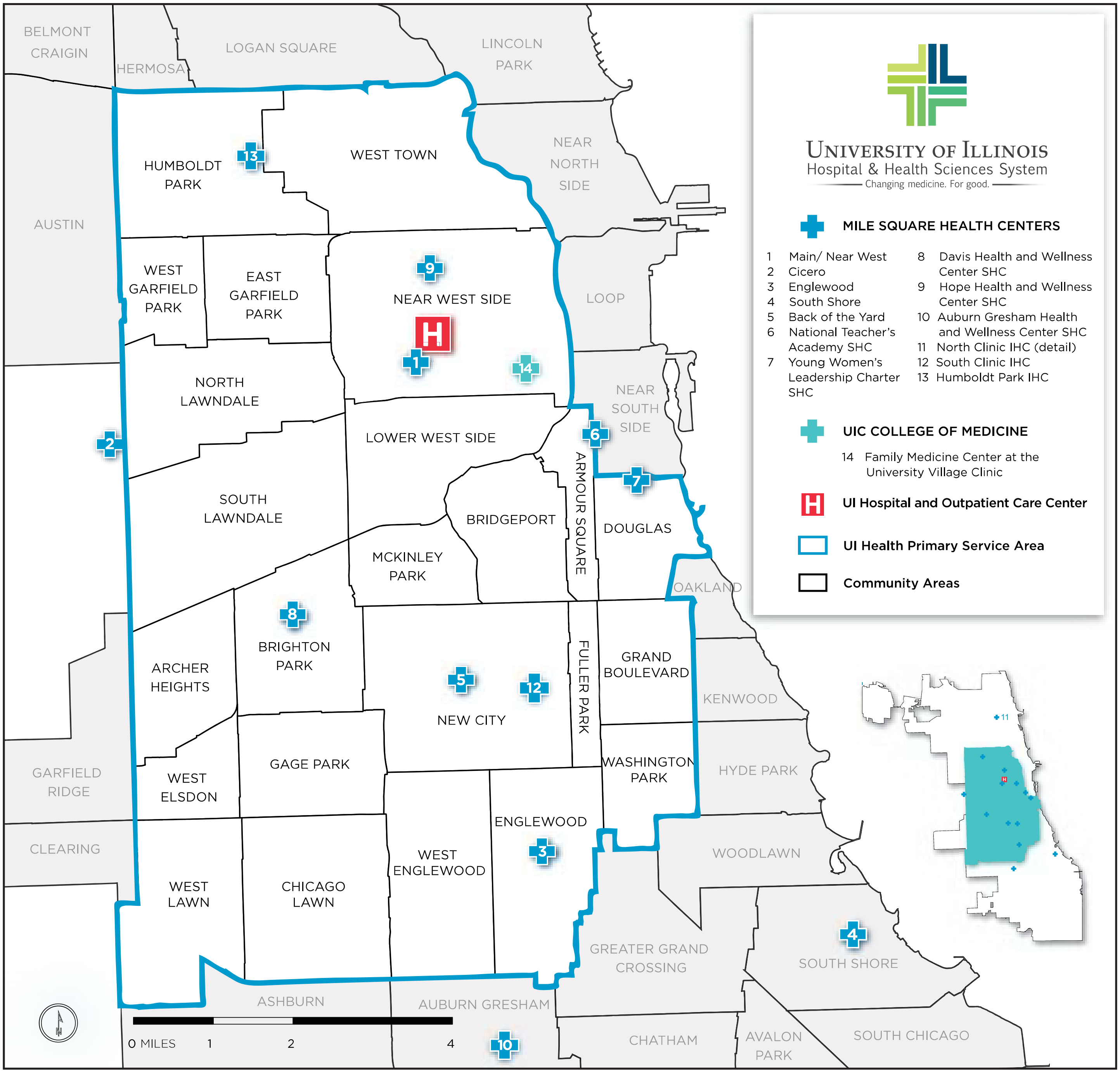

Clinic information on back ▶

Mile Square Health Centers

1. Main/Near west

1220 South Wood St.
Chicago, IL 60608
312.996.2000

Mon–Fri 9am-5pm
Sat 8am-noon
Sun Closed

2. Cicero

Hawthorne Works Shopping Center
4745-51 West Cermak Rd.
Cicero, IL 60804
708.656.4766

Mon 9am-5pm
Tues 9:30am-5:30pm
Wed 9am-5pm
Thurs 9am-5pm
Fri 9am-5pm
Sat + Sun Closed

3. Englewood

641 West 63rd St.
Chicago, IL 60621
312.413.4070

Mon-Fri 9am-5pm
Sat + Sun Closed

4. South Shore

7131 S. Jeffery Blvd.
Chicago, IL 60649

Mon + Tues 9am-5pm
Wed 9:30am-5:30pm
Thurs + Fri 9am-5pm
Sat + Sun Closed

5. Back of the Yards

4630 South Bishop St.
Chicago, IL 60609
773.523.2615

Mon + Tues 8:30am-5pm
Wed 9:30am-5:30pm
Thurs + Fri 8:30am-5pm
Sat 8:30am-noon
Sun Closed

6. National Teachers Academy School-based Health Center

55 West Cermak Rd.
Chicago, IL 60616
312.326.4472

7. Young Women's Leadership Charter School-based Health Center

2641 South Calumet Ave.
Chicago, IL 60616
312.949.0277

8. Davis Health and Wellness Center School-based Health Center

3050 West 39th Place
Chicago, IL 60632
773.376.8008

9. Hope Health and Wellness Center School-based Health Center

1628 West Washington Blvd.
Chicago, IL 60612
312.226.3288

10. Auburn Gresham Health and Wellness Center School-based Health Center

8131 S May St.
Chicago, IL 60620
312.355.5801

11. North Integrated Health Care Clinic (detail)

4221 North Lincoln Ave.
Chicago, IL 60618
773.537.3950
773.537.3020

Mon-Fri
9am-noon, 1pm-5pm

12. South Integrated Health Care Clinic

734 West 47th St.
Chicago, IL 60609
773.537.3960
773.537.3238

Mon-Fri
9am-noon, 1pm-5pm

13. Humboldt Park Integrated Health Care Clinic

3240 West Division St.
Chicago, Illinois 60651
312.413.7425

Mon-Fri
9am-noon, 1pm-5pm

UIC College of Medicine

14. Family Medicine Center at the University Village Clinic

722 West Maxwell St.
Suite 235
Chicago, IL 60607

Mon 8am-5pm
Tues 8am-9pm
Wed 9:30am-5pm
Thurs 8am-9pm
Fri 7am-5pm
Sat 9am-Noon

2 Keep track of doctor's visits + medications

Bring this information when you talk to the pharmacist, your doctor or your peer coach.

This information is for your follow-up period.
Get updated information from your primary doctor.

Visit http://hospital.uillinois.edu/, then click **"For Patients + Visitors,"** then **"Patient Information,"** then **"My UI Health."**

What's inside

2A Appointment information

2B Medication routine

2C Your healthcare information

This information will help you...

- keep track of your follow-up doctor's appointments
- maintain your new medication routine

2A Appointment information for May 2016

Notes

Su	M	T	W	Th	F	Sa
1	2	3	4	5	6	7
8	9	10	11	12	13	14
15	16	17	18	19	20	21
22	23	24	25	26	27	28
29	30	31				

Support groups

**Sickle Cell Center**	312.996.5267
**Mended Hearts Support Group** for heart conditions	312.926.2070
**Stroke Support Group**	312.996.3700
**COPD Foundation**	866.316.COPD (2673)
**University of Illinois Cancer Center**	312.355.1625

Date of birth:

2A Appointment information for May 2016 continued

Notes

Su	M	T	W	Th	F	Sa
1	2	3	4	5	6	7
8	9	10	11	12	13	14
15	16	17	18	19	20	21
22	23	24	25	26	27	28
29	30	31				

2A Appointment information for June 2016

Notes

Su	M	T	W	Th	F	Sa
			1	2	3	4
5	6	7	8	9	10	11
12	13	14	15	16	17	18
19	20	21	22	23	24	25
26	27	28	29	30		

Healthy eating

3 tips for healthy eating:

- Eat more of certain foods and nutrients found in fruits, vegetables, whole grains, low-fat dairy products + seafood.
- Eat fewer foods with sodium, saturated fats, trans fats, cholesterol, added sugars + refined grains.
- Balance calories with physical activity to manage weight.

2A Appointment information for June 2016 continued

Notes

Su	M	T	W	Th	F	Sa
			1	2	3	4
5	6	7	8	9	10	11
12	13	14	15	16	17	18
19	20	21	22	23	24	25
26	27	28	29	30		

Date of birth:

2A Appointment information for July 2016

Notes

Su	M	T	W	Th	F	Sa
					1	2
3	4	5	6	7	8	9
10	11	12	13	14	15	16
17	18	19	20	21	22	23
24	25	26	27	28	29	30
31						

Control your blood pressure

- Take medication as prescribed.
- Follow a healthy diet.
- Reduce the salty foods you eat.
- Maintain a healthy weight.
- Get tested for sleeping problems.
- Reduce stress.

Date of birth:

2A Appointment information for July 2016 continued

Notes

Su	M	T	W	Th	F	Sa
					1	2
3	4	5	6	7	8	9
10	11	12	13	14	15	16
17	18	19	20	21	22	23
24	25	26	27	28	29	30
31						

2A Appointment information for August 2016

Notes

Su	M	T	W	Th	F	Sa
	1	2	3	4	5	6
7	8	9	10	11	12	13
14	15	16	17	18	19	20
21	22	23	24	25	26	27
28	29	30	31			

Quit smoking for good!

Stay motivated

Make a list of reasons to quit and carry it with you.

Set a date

Preparing for your quit date is key to starting a new, smoke-free life.

Find support

Spend time with non-smokers and ex-smokers.

Tobacco Treatment Center 312.996.3300

2A Appointment information for August 2016 continued

Notes

Su	M	T	W	Th	F	Sa
	1	2	3	4	5	6
7	8	9	10	11	12	13
14	15	16	17	18	19	20
21	22	23	24	25	26	27
28	29	30	31			

2A Appointment information for
September 2016

Notes

Su	M	T	W	Th	F	Sa
				1	2	3
4	5	6	7	8	9	10
11	12	13	14	15	16	17
18	19	20	21	22	23	24
25	26	27	28	29	30	

What is medicaid?

Medicaid is a free or low-cost insurance plan based on income.

You are eligible if you earn an income of less than $15,800 for an individual or $32,500 for a family of 4.

PARTNER

Date of birth:

University of Illinois Hospital & Health Sciences System
Changing medicine. For good.

2A Appointment information for September 2016 continued

Notes

Su	M	T	W	Th	F	Sa
				1	2	3
4	5	6	7	8	9	10
11	12	13	14	15	16	17
18	19	20	21	22	23	24
25	26	27	28	29	30	

2A Appointment information for October 2016

Notes

Su	M	T	W	Th	F	Sa
						1
2	3	4	5	6	7	8
9	10	11	12	13	14	15
16	17	18	19	20	21	22
23	24	25	26	27	28	29
30	31					

Prevent the flu

- Get a flu shot every year in the fall or winter months.
- Wash your hands often and well.
- Avoid being near people who are sick.
- Eat a healthy diet.

Date of birth:

2A Appointment information for October 2016

Notes

Su	M	T	W	Th	F	Sa
						1
2	3	4	5	6	7	8
9	10	11	12	13	14	15
16	17	18	19	20	21	22
23	24	25	26	27	28	29
30	31					

Date of birth:

2B *Keep track of doctor's visits + medications*

Medication routine

This information is a guide only. Be sure to read instructions from your doctor and on the medication bottle.

Bring this information when you talk to the pharmacist, your doctor or your peer coach.

Ask your doctor if you should still be taking each of these medications and what each medication is taken for.

Medication name	Morning	Noon	Evening	Bedtime	As needed	Notes
	☐	☐	☐	☐	☐	
	☐	☐	☐	☐	☐	
	☐	☐	☐	☐	☐	

Medication name	Morning	Noon	Evening	Bedtime	As needed	Notes
	☐	☐	☐	☐	☐	
	☐	☐	☐	☐	☐	
	☐	☐	☐	☐	☐	
	☐	☐	☐	☐	☐	
	☐	☐	☐	☐	☐	

Date of birth:

Medication name	Morning	Noon	Evening	Bedtime	As needed	Notes
	☐	☐	☐	☐	☐	
	☐	☐	☐	☐	☐	
	☐	☐	☐	☐	☐	
	☐	☐	☐	☐	☐	
	☐	☐	☐	☐	☐	

PARTNER

Date of birth:

University of Illinois
Hospital & Health Sciences System
Changing medicine. For good.

Medication name	Morning	Noon	Evening	Bedtime	As needed	Notes
	☐	☐	☐	☐	☐	
	☐	☐	☐	☐	☐	
	☐	☐	☐	☐	☐	
	☐	☐	☐	☐	☐	
	☐	☐	☐	☐	☐	

Date of birth:

Medication name	Morning	Noon	Evening	Bedtime	As needed	Notes
	☐	☐	☐	☐	☐	
	☐	☐	☐	☐	☐	
	☐	☐	☐	☐	☐	
	☐	☐	☐	☐	☐	
	☐	☐	☐	☐	☐	

Medication name	Morning	Noon	Evening	Bedtime	As needed	Notes
	☐	☐	☐	☐	☐	
	☐	☐	☐	☐	☐	
	☐	☐	☐	☐	☐	
	☐	☐	☐	☐	☐	
	☐	☐	☐	☐	☐	

PARTNER

Date of birth:

University of Illinois
Hospital & Health Sciences System
Changing medicine. For good.

Medication name	Morning	Noon	Evening	Bedtime	As needed	Notes
	☐	☐	☐	☐	☐	
	☐	☐	☐	☐	☐	
	☐	☐	☐	☐	☐	
	☐	☐	☐	☐	☐	
	☐	☐	☐	☐	☐	

Date of birth:

Medication name	Morning	Noon	Evening	Bedtime	As needed	Notes
	☐	☐	☐	☐	☐	
	☐	☐	☐	☐	☐	
	☐	☐	☐	☐	☐	
	☐	☐	☐	☐	☐	
	☐	☐	☐	☐	☐	

Date of birth:

Medication name	Morning	Noon	Evening	Bedtime	As needed	Notes
	☐	☐	☐	☐	☐	
	☐	☐	☐	☐	☐	
	☐	☐	☐	☐	☐	
	☐	☐	☐	☐	☐	
	☐	☐	☐	☐	☐	

Medication name	Morning	Noon	Evening	Bedtime	As needed	Notes
	☐	☐	☐	☐	☐	
	☐	☐	☐	☐	☐	
	☐	☐	☐	☐	☐	
	☐	☐	☐	☐	☐	
	☐	☐	☐	☐	☐	

Date of birth:

2C *Keep track of doctor's visits + medications*
Your healthcare information

Bring this information when you talk to the pharmacist, your doctor or your peer coach.

For physician use:

Hospitalization summary

Patient name:

Medical record #:

Date of birth:

Admission date:

Discharge date:

Primary discharge diagnosis:

For patient reference:

Insurance

Insurance type:

Insurance #:

Insurance phone #:

Insurance care coordinator:

Pharmacy

Pharmacy:

Address:

Pharmacy phone #:

Hours:

3 Find support in your community

Have this information when you talk to your navigator or peer coach.

What's inside

3A Resources for you

3B Community resources

This information will help you...

- gain access to resources you need
- keep track of resources you are in the process of getting
- get the most out of your navigator visit and peer coach phone calls

3A *Find support in your community* Resources for you

Category	Resource + description	Contact information

Date of birth:

Category	Resource + description	Contact information

3B *Find support in your community*
General resources

**Transportation**	**First Transit**<br>*Non-emergency transportation services for patients with Medicaid*	**Contact information**<br>877.725.0569<br>**Mon-Fri** 8am-5pm
	**ADA Paratransit**<br>*For patients with disabilities*	312.663.HELP (4357)<br>TTY: 312.913.3122
**Childcare**	**Children's programs at Greater Food Repository**	773.247.3663
**Housing**	**Chicago Department of Family and Support Services**	Garfield Center<br>312.746.5400<br>North Side Center<br>312.744.2580<br>South Chicago Center<br>312.747.0331<br>King Center (South side)<br>312.747.2300<br>Englewood (South side)<br>312.747.0200<br>Trina Davila (North side)<br>312.744.2014
	**Department of Human Services**	312.746.5400
	**CEDA Home Energy Bill Payment Assistance**	800.571.2332<br>call for a referral CEDA intake site near you

Food	**Greater Chicago Food Depository**<br>*Ask for SNAP Outreach Team*	773.247.FOOD (3663)<br>**Mon-Fri** 8:30am-5pm
	**Illinois Hunger Coalition**	800.359.2163
Healthcare	**National Tobacco Quit Line**	1.800.QUIT.NOW (1.800.784.8669)
	<u>UI Health</u>	
	Dental + Oral Care clinic	312.996.7555
	Eye + Ear Infirmary	312.996.6590
	Family Medicine Center	800.866.CARE
	Neuropsychiatry and Psychology	312.996.7723
	Hospital + Emergency Room	800.866.CARE
	Sickle Cell Center	312.413.8666
	Heart Failure Program	312.413.8443
	Lung Health Program	312.996.3300
	Tobacco Treatment Center	312.996.3300
	Cancer Center	<u>New patients</u><br>866.600.CARE<br><br><u>Existing patients</u><br>312.355.1625 (Cancer center)<br>312.355.4300 (Surgery Center)
Other needs	Non-emergency police	311

4 Take control of your care!

Take notes!

Keep a record of questions, concerns, important information from the doctor or pharmacist, and any challenges you are having getting follow-up appointments or resources.

Have these available when you talk with your peer coach.
